# Feasibility and preliminary validity evidence for remote video-based assessment of clinicians in a global health setting

**DOI:** 10.1371/journal.pone.0220565

**Published:** 2019-08-02

**Authors:** Katherine A. Smith, Segolame Setlhare, Allan DeCaen, Aaron Donoghue, Janell L. Mensinger, Bingqing Zhang, Brennan Snow, Dikai Zambo, Kagiso Ndlovu, Ryan Littman-Quinn, Farhan Bhanji, Peter A. Meaney

**Affiliations:** 1 Children’s Hospital of Philadelphia, Philadelphia, Pennsylvania, United States of America; 2 American Heart Association, Kweneng, Botswana; 3 University of Alberta, Edmonton, Alberta, Canada; 4 Perlman School of Medicine, University of Pennsylvania, Philadelphia, Pennsylvania, United States of America; 5 Drexel University, Philadelphia, Pennsylvania, United States of America; 6 B-Line Medical Charitable Fund, Washington D.C., United States of America; 7 Botswana-UPenn Partnership, Gaborone, Botswana; 8 University of Botswana, Gaborone, Botswana; 9 McGill Department of Pediatrics, Montreal, Quebec, Canada; 10 Stanford University School of Medicine, Stanford, California, United States of America; University of North Carolina at Chapel Hill, UNITED STATES

## Abstract

**Background:**

Serious childhood illnesses (SCI), defined as severe pneumonia, severe dehydration, sepsis, and severe malaria, remain major contributors to amenable child mortality worldwide. Inadequate recognition and treatment of SCI are factors that impact child mortality in Botswana. Skills assessments of providers caring for SCI have not been validated in low and middle-income countries.

**Objective:**

To establish preliminary inter-rater reliability, validity evidence, and feasibility for an assessment of providers who care for SCI using simulated patients and remote video capture in community clinic settings in Botswana.

**Methods:**

This was a pilot study. Four scenarios were developed via a modified Delphi technique and implemented at primary care clinics in Kweneng, Botswana. Sessions were video captured and independently reviewed. Response process and internal structure analysis utilized intra-class correlation (ICC) and Fleiss’ Kappa. A structured log was utilized for feasibility of remote video capture.

**Results:**

Eleven subjects participated. Scenarios of Lower Airway Obstruction (ICC = 0.925, 95%CI 0.695–0.998) and Hypovolemic Shock from Severe Dehydration (ICC = 0.892, 95%CI 0.596–0.997) produced excellent ICC among raters while Lower Respiratory Tract Infection (LRTI, ICC = 0, 95%CI -0.034–0.97) and LRTI + Distributive Shock from Sepsis (0.365, 95%CI -0.025–0.967) were poor. Oxygen therapy (0.707), arranging transport (0.706), and fluid administration (0.701) demonstrated substantial task reliability.

**Conclusions:**

Initial development of an assessment tool demonstrates many, but not all, criteria for validity evidence. Some scenarios and tasks demonstrate excellent reliability among raters, but others may be limited by manikin design and study implementation. Remote simulation assessment of some skills by clinic-based providers in global health settings is reliable and feasible.

## Introduction

Worldwide, child mortality has fallen 52% over the last 25 years [1, 2]. Despite these improvements, serious childhood illnesses (SCI), defined as severe pneumonia, severe dehydration, sepsis, and severe malaria, remain major contributors to mortality in children [3]. Previous studies have demonstrated that situational awareness and operational performance of clinic-based providers in low and middle income countries (LMICs) are often poor and have significant variability [4–7].

In Botswana, 83% of people live within 8km of a health facility (100% within 15km) [8–10]. The estimated doctor/population ratio is 1:550 and nurse/population ratio is 1:80. According to the latest Health Statistics Report, diarrhea and pneumonia were the leading two causes of death for infants and children less than 5 years of age. In 2014, diarrhea accounted for 21.4% of under-five mortality and pneumonia accounted for 16.7% of under-five mortality, with 140 and 114 child deaths respectively [11]. Botswana’s access to quality health care ranks 44^th^ out of 49 countries with comparable sociodemographic index, and treatment of diarrhea and lower respiratory infections are two of the lowest subscores among all conditions [3]. Additionally, a standardized mortality audit revealed that one third of in-hospital pediatric deaths in Botswana occurr within the first 24 hours of admission, an indication that children arrive to the hospital critically ill [12].

In Botswana, access to quality services for treatment of LRTI and diarrhea have lagged in comparison to the provision of preventive services [3]. Inadequate recognition and treatment of diarrhea and LRTI in clinics were major modifiable factors contributing to child mortality [12]. Saving Children’s Lives (SCL) is a training program focused on improving performance of healthcare providers (doctors, nurses) in caring for seriously ill children [13]. Implementation of SCL in the Kweneng district of Botswana was associated with a 41% reduction of in-hospital child mortality at the district hospital after the first year of implementation [14].

Although the burden of child deaths occur in LMICs, skills assessments with standardized feedback have primarily been validated in high income countries [13, 15–17]. No validated skills assessment exists for clinic-based providers caring for seriously ill children in LMICs [18]. The lack of remote assessment capabilities limits provider training to urban centers and developed countries and is prohibitive to continuous training. Current training paradigms also require international faculty to be present and local providers to take considerable time away from the facilities in which they work. Better assessment tools used in the real world may help to sustain skills.

It is unknown if a difference in assessment skills exists between raters more experienced in course content and educational constructs compared with raters more experienced with learner’s work environments. It is also unknown if remote assessment using simulation software and a cloud-based server is feasible in low-resource settings. These gaps inhibit the development of a high-quality, sustainable program within a healthcare system to assess and improve provider skills in treating SCI. Establishment of validity evidence to support a scoring instrument and remote video capture will help address these gaps.

The primary objective of this pilot study was to assess the preliminary inter-rater reliability among expert raters on a novel assessment tool of provider clinical performance in a simulated setting. We hypothesized that assessments of clinic-based providers who care for SCI are reliable when providers are assessed by experts. Secondary objectives were to evaluate the construct validity of the assessment tool, to determine the inter-rater reliability in scoring between in-country instructors and international raters, and to demonstrate feasibility of telementoring.

## Methods

### Study design

We conducted a prospective observational cohort study of providers working at health posts, clinics, and hospitals in the Kweneng District of Botswana in June-August 2017. Any clinic-based provider who was an SCL instructor or instructor candidate was eligible to participate. Eligible participants were identified through training records, and all participants provided voluntary informed consent prior to participation. Scenarios were administered in random order using a block randomization schedule, with one scenario per participant. Five investigators served as the raters for this study and independently scored each recorded case. All scenarios were captured using the SimCapture UltraPortable (B Line Medical, Washington, D.C., USA), a mobile video/audio capture device that provides user management, curriculum management, assessment, and reporting capabilities. This was managed on a windows-based laptop, with two cameras using the B Line software.

### Assessment tool development

Four scenarios were developed: Lower Respiratory Tract Infection (LRTI), Lower Airway Obstruction (LAO), Hypovolemic Shock from Severe Dehydration (HSSD), and LRTI with Distributive Shock from Sepsis (LRTI+DSS) (S1, S2, S3 and S4 Appendices). These scenarios were based on the six cases used in the Saving Children’s Lives course [13], and the SCL Critical Skills Checklist was used to identify tasks that could distinguish novice from expert performance (S5 Appendix). A 1 year old, 10kg infant was used in each simulated scenario.

Scoring definitions for each task were determined by consensus of the five investigators through a four-cycle modified Delphi process [19–24]. The investigative team consisted of two pediatric critical care medicine physicians, two dual-trained pediatric emergency and critical care medicine physicians, and a former senior registered nurse at the Kweneng District Hospital Accident and Emergency department. Among them is a current member and past chairs of the American Heart Association Emergency Cardiovascular Care Pediatric Subcommittee and Education Subcommittee, members of the SCL Educational Design Committee, Director of SCL, and SCL Site Coordinator in Kweneng. These investigators also served as the raters reviewing each participant’s performance, with four international raters from the United States and Canada, and one local rater from Botswana.

The proposed assessment tool underwent four major revisions during the refinement process: one round of email, two phone conferences, and one round of initial testing. Based on previous instruments, it was determined that individual tasks would each be scored trichotomously [15, 25] and a 5-point holistic rating was added to increase rater discrimination [26]. All four cases had an initial test of the assessment tool with non-study participants by all raters. Definitions were further refined to ensure inclusivity and agreement among investigators (S6 Appendix). A table of acceptable medication dosages, routes of administration, and duration of administration was created using local references (S7 Appendix). Raters’ participation in the development, initial testing, and revision of the assessment tool served as training on use of the assessment tool.

### Scenario implementation

Using the SimCapture UltraPortable, two standardized video angles captured the provider’s actions but not their face (S8 Appendix). Sessions were facilitated by the SCL Site Coordinator and introduced to participants using a standardized script (S9 Appendix). Available supplies were displayed to the camera as a written list at the start of each session. Simulations were five minutes in duration, with an audible timer signaling the end of simulation, and were followed immediately by debriefing between the facilitator and participant. After sessions were recorded, devices were connected to the network for video centralization. Videos were accessed on the virtual centralization server by raters via password-protected URL links, and assessments were completed while viewing the pre-recorded videos.

### Statistical approach

Mean scores from five raters were calculated to show the performance for each participant. Median and interqutile ranges of the mean scores were presented by instructor status (instructors versus instructor candidates), as well as by each senario.

The inter-rater reliability of overall competency was assessed through intra-class correlation (ICC) based on a single-rating, two-way random-effects model. This model was chosen because the study was fully crossed, and a single-rating was used to assess the overall competency of the provider in practice. ICC estimates were calculated using the ‘IRR’package, R 3.2.5. The ICC of overall score for each scenario was calculated among all five raters, including the four international raters and one local rater, as well as among international raters only. We defined, a-priori, the strata of ICC to be: ICC < 0.40 = poor agreement; 0.40–0.59 = fair; 0.60–0.74 = good; and 0.75–1.00 = excellent agreement [27–29].

To test the item-level inter-rater reliability and identify items that are difficult to rate, Fleiss’ Kappa Coefficient was computed to assess the agreement among all raters on each item of the scenarios. Strata for kappa values were also defined a-priori, as: ≤0 = no agreement; 0.01–0.20 = none to slight; 0.21–0.40 = fair; 0.41–0.60 = moderate; 0.61–0.80 = substantial; and 0.81–1.00 = almost perfect agreement [30]. Items with a kappa of ≤0.40 were identified as “difficult to rate” and will be targeted for future review and refinement.

### Feasibility assessment

The anticipated technical requirements for the project included SimCapture Virtual Centralization Server (hosted in AWS Cloud), 2 SimCapture UltraPortable Devices, static connection to the internet, local data upload of 4 GB/month, and a secure storage location for devices at the district hospital. The anticipated workflow of the local study team included travelling to multiple clinics per day and recruiting one to two participants per site. The investigative team utilized a running log to capture technical issues and logistical challenges during the study.

### Ethics/IRB considerations

The study was approved by the Institutional Review Boards at the Botswana Ministry of Health (Reference Number HPDME 13/18/1) and the University of Pennsylvania (IRB Protocol 829892). Voluntary written informed consent was obtained from all subjects prior to participation.

## Results

**Fig 1:**
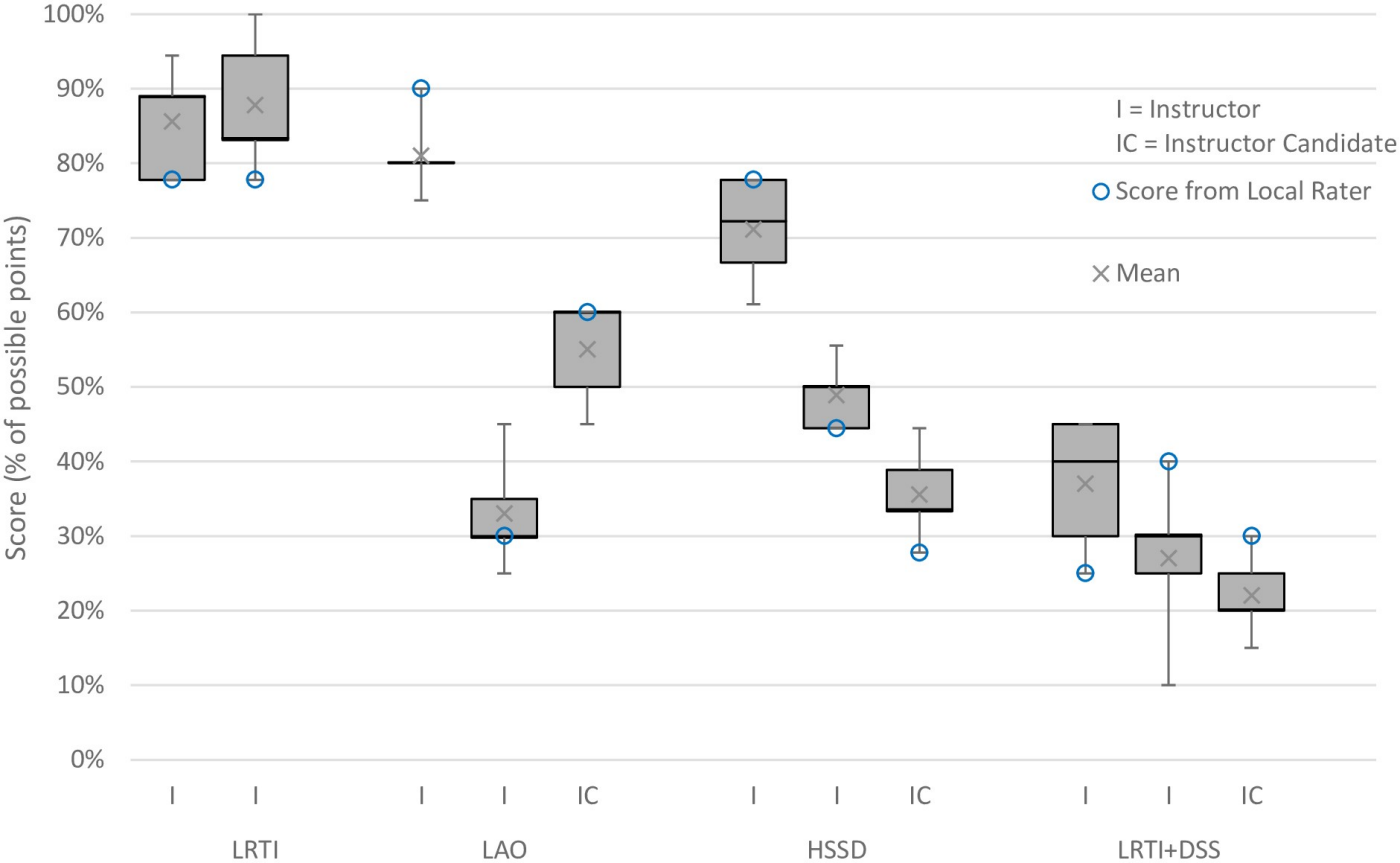
Median (IQR) scores for four scenarios by participant. Note: Each bar represents one participant’s scores by all five raters (international + local), with higlighted scoring by the local rater.

This pilot study assessed eleven participants: eight instructors and three instructor candidates. Participant scores, averaged from all five raters, ranged from 0.22–0.80, while participant scores from individual raters ranged from 0.10–1.00 (Fig 1).

The median score on LRTI was 0.87, LAO was 0.55, HSSD was 0.49, and LRTI+DSS was 0.27 (Table 1). The median instructor score was 0.60 and median instructor candidate score was 0.36 (Table 2).

**Table 1:** Variability in difficulty between scenarios.

Case	n	Averaged total score median (IQR)
LRTI	2	0.87 (0.87–0.88)
LAO	3	0.55 (0.33–0.81)
HSSD	3	0.49 (0.36–0.71)
LRTI+DSS	3	0.27 (0.22–0.37)

**Table 2:** Preliminary construct validity—Instructor versus instructor candidate.

Instructor Status	n	Averaged total score median (IQR)
Instructor	8	0.60 (0.35–0.83)
Instructor Candidate	3	0.36 (0.22–0.55)

### Reliability of overall competency and reliability among raters

Among international raters, cases for LAO (n = 3) and HSSD (n = 3) produced excellent reliability, with ICCs of 0.917 and 0.884 respectively (Table 3). With the inclusion of the local rater, reliability remained excellent: LAO ICC 0.925 and HSSD ICC 0.892. The LRTI+DSS case (n = 3) had good agreement among international raters (ICC 0.677), but fell to poor with the inclusion of the local rater (ICC 0.365). The LRTI case (n = 2) had poor agreement (ICC 0) among both international raters only, as well as among all raters.

**Table 3:** ICC with and without local rater.

Case	n	ICC (International Raters only, n = 4)	95% CI	ICC (International + Local Rater, n = 5)	95% CI
LRTI	2	n/c*	n/c	n/c	n/c
LAO	3	0.917	(0.633, 0.998)	0.925	(0.695, 0.998)
HSSD	3	0.884	(0.513, 0.997)	0.892	(0.596, 0.997)
LRTI+DSS	3	0.677	(0.149, 0.988)	0.365	(-0.025, 0.967)

*Not able to calculate LRTI ICC due to small n (n = 2)

### Reliability of items

Items that demonstrated substantial to moderate reliability among all five raters using Fleiss’s Kappa Coefficient were (in descending order): oxygen therapy 0.707, arrange transport 0.706, fluid bolus 0.701, IV placement 0.578, IO placement 0.522, reassessment 0.456, applies monitors 0.432, and general assessment 0.406. Items that were difficult to rate (fair to no agreement) were: medication administration 0.322, patient assessment 0.273, and nebulizer therapy -0.05.

### Feasibility

We tracked technical and logistical solutions to challenges encountered during this study (Table 4). Solutions included providing an independent continuous internet connection for the program’s office at the hospital, instituting a daily UltraPortable device rotation, defining standardized camera angles, and video recording the list of available supplies at the start of each scenario. The independent office internet overcame two obstacles: it avoided the variability in the hospital connection as well as the variability of cellular network strengths in different clinic locations. Device rotation overcame the latent bandwidth/data transfer speeds and inconsistent power sources at clinics, allowing one to capture sessions while the other recharged and centralized data with the cloud-based server. In addition, we utilized a car charger so that re-charging was possible while en route between clinics.

**Table 4:** Feasibility log.

Challenge	Consequence	Solutions Utilized
Technical		
Inconsistent power source in rural clinics	Limited remote daily recording time/Upload time/Risk of data loss	Added rotation / schedule for the two SimCapture UltraPortable devices (one in use and one centralizing at a time) to increase battery charge capacity to capture sessions Use of SS Hard disks to minimize data loss if laptop left on during rural transport Car charger to charge laptop and cell phone during travel between clinics.
Cellular service availability differed by carrier in different clinic sites.	Limited remote daily recording time/Upload time/Risk of data loss	Data upload when return to district hospital. *Future*: *Determine best bandwidth for cellular service on initial assessment of clinic sites*, *quarterly (speedtest*.*net) (Wireless carrier A for urban*, *Wireless carrier B for rural)* *Pay as you go for all local carriers/anticipate changing outages*
District hospital internet connection varied significantly with time of day (75kps day– 2 Mbps night).	Local instructors unable to view session once uploaded to server	Created workflow for local users to allow for downloading and locally viewing the session videos from virtual server. Provided continuous internet connection for district hospital office to facilitate transmission independent of hospital’s network.
Bandwidth / Transfer speeds much more latent than in North America	HTTP header transmission failing during session upload attempts Caused video transfer failure initially from district hospital to virtual centralization server Time required to upload/centralize video from local SimCapture to virtual centralization server far exceeded traditional expectation	Reduced HTTP packet header size to ensure successful transmission Data upload overnight (requires daily return to district hospital) Added rotation / schedule for the two SimCapture UltraPortable devices (one in use and one centralizing at a time) to increase capacity to capture sessions Requires secure space for storage to have reliable/persistent internet connection
Cannot guarantee static IPs for these devices at SLH	SimCapture units needed to be configured with hostnames. As a result, not possible to pass through to local SimCapture device from the centralization server to view session, needed to wait for centralization of video to view remotely	Developed on site workflow to minimize backlog of data to be uploaded overnight.
Annotations of session not able to be associated with individuals	Raters not able to utilize annotation function as not able to blind other raters.	*Future*: *software design to allow annotations to be assigned at individual level*, *rather than session level*
Session Playback requires Flash Player	Limits playback to flash player compatible devices, limited tablet debriefing	*Future*: *software to use universally acceptable platforms to allow portable devices for review and annotate*, *and submit assessments of sessions*
Logistical		
Designated Local IT support significant distance away	Technical device malfunction/breakage prohibits data capture and halts program	Had 2 devices for rotation of recordings and uploading/repairs if necessary Post-hoc engagement DHMT IT support for troubleshooting *Future*: *Have 3 devices for needs anticipated in the field (1 working*, *1 uploading*, *1 repair)* *Schedule on-site maintenance/equipment rotation to central support to overlap with other projects being supported* *Formally engage/collaborate with Ministry of Health/DHMT IT support*
Missing/Non-functioning clinic equipment for use in scenario	Reinforcing/teaching incorrect Diagnostics &Therapeutics	Either practice scenario without expected equipment (limited effect) or with substitute (requires further integration of learning after scenario) List of supplies available in clinician’s setting shown to camera at the start of each session *Future*: *Standardize list of commonly missing critical equipment*: *Real time feedback to local clinic supervisor and DHMT* *Partner with DHMT to bring replacements*, *restock back at DHMT*. *Generate acceptable alternatives of Diagnostic and therapeutics (DHMT*, *MoH approved)*
Video not optimally positioned to capture session	Raters unable to see actions/inaction	Standardized camera set up *Future*: *reduce need for verbal verification of knowledge and skills through development of a more responsive manikin*
Participants speak in local dialect (Setswana)	International faculty cannot understand, may not reflect provider knowledge	Had facilitator repeat back/use closed loop communication of what they said in English *Future*: *reduce need for verbal verification of knowledge and skills through develop more responsive manikin*
Varied perception of correct/expected diagnostics and therapeutics at the clinic level	Reinforcing/teaching incorrect Diagnostics &Therapeutics	Created list of correct medications with references used with page numbers (national drug formulary, treatment guides, British formula) *Future*: *verify with Cluster*, *DHMT and MoH department*, *leadership approval*
Local study team unable to travel to clinics and enroll participants at the frequency anticipated	Limited enrollment of participants	*Future*: *Reduce study team responsibilities/increase study team size*

A standardized camera set-up and video capture of available supplies at the initiation of Ultraportable data capture addressed the variability of resources available between clinics and days. This allowed the raters to evaluate the clinician on their performance with what resources they actually had available, rather than what tools they should have had in an ideal setting. A master medication list addressed any rater perceptions on what the correct treatment should be at the clinic level.

## Discussion

This study provides preliminary evidence for validity of a video assessment tool allowing remote expert assessment of provider skills in LMIC clinics. Our hypothesis was that a low-stakes assessment of clinic-based providers caring for seriously ill children is reliable when providers are assessed by experts. We discuss the results using a framework of the five commonly accepted sources of validity evidence [31, 32].

Scenario development by experts in education and pediatric resuscitation science provides content validity evidence. Scenarios were based on demographic data, local epidemiology, and the SCL training program blueprint for Botswana [12, 13]. The expected responses were tailored to the work setting of each provider so that differences did not impact scores, and scenarios were monitored closely to ensure all participants experienced the same core objectives. The use of a modified Delphi approach also provides content–expert agreement. Our results show that a different rater, trained on the same case, rates provider performance similarly.

The response process of scenarios provides preliminary evidence of validity. Scenario introductions were standardized, and facilitator responses for each case were defined. Rater data was collected remotely via simulation software which provides basic quality control of rating data and prevents scoring contamination by other raters. The rationale for both a global rating and specific tasks is provided. While some individual tasks were consistently scored across all cases, others demonstrated lower Fleiss’ kappa values. Inconsistency among raters for these tasks may represent limitations by low enrollment or intrinsic in the equipment used for the simulation.

The statistical relationship between and among other measured traits of raters and participants provides preliminary validity evidence of internal structure. LAO and HSSD cases produced excellent ICCs, indicating reliability of evaluation between all raters. Local and international raters scored participants similarly for three of the four cases, which indicates that local providers may reliably evaluate skills in caring for SCI using this tool. LRTI+DSS demonstrated good reliability among international raters, but reliability was poor with the inclusion of the local rater. This may be due to rater experience, orientation to the scenario, or that tasks required greater inference by the raters due to poor fidelity of the simulated scenario to training or practice. Alternatively, the local rater, who also served as the faciliatator of the scenarios, may have had better direct observation and understanding of any local dialect than the interational raters restricted to recorded audio and video. LRTI demonstrated poor reliability among all raters, which could also be due to poor fidelity of the simulated scenario, but may be due to low numbers of participants completing the scenario.

LRTI+DSS was anticipated and shown to be the most difficult case, likely due to the complexity of the condition, and LRTI was shown to be the easiest of the four scenarios. The constructs assessed in the LRTI+DSS scenario were more complex, representing a multiple problem scenario, which was anticipated to result in lower scores than single problem scenarios. In addition, we attempted to discriminate between instructors and instructor candidates, hypothesizing that instructors would outperform instructor candidates. Instructor scores indeed trended higher than instructor candidates in nearly all cases where a comparison could be made. (*LRTI did not have any instructor candidates to make the comparison.) This highlights potential evidence for the test’s construct validity, as instructors have likely had more engagement with the content being measured by the designed evaluations than instructor candidates.

As there is no existing measure with well-known characteristics against which to compare our new assessment, we were not able to provide validity evidence of the relationships to other variables. There is no criterion gold-standard test against which the current exam of SCL training can be compared.

Consequential validity was appropriate, as the assessment was designed to be low-stakes. There was no risk of the participant losing their job, SCL instructor status, or further opportunities for education based on their performance. There was a debriefing immediately after the scenario to provide feedback to the participant, reinforcing actions the participant did well and providing recommendations for improving future care. The participant was also given the opportunity to ask questions. Potential consequences of having better trained and assessed instructors may be better training of frontline providers and therefore better patient care and improved patient outcomes. Additionally, remote assessment may allow for more frequent training and learning opportunities that will maintain skills more effectively than intermittent bolus training.

This study demonstrated the feasibility of remote simulation skills assessment in LMICs. While implementing this study, existing hardware was adapted to complete the study, and many barriers were overcome by strengthening logistic support. Reducing the size of data packets allowed transmission over an unstable network. Further, use of three devices allowed one to be used for training, one to charge and sync with the server, and one to be repaired. However, playback of video-recorded sessions for evaluation after sync required a flash player, limiting access to raters with poor internet connection. A tablet/phone-based platform may greatly reduce the barrier of technology, but currently none exist.

### Significance

The long-term goal of this study is to improve the quality of care for seriously ill children. Facilitating regular simulations of seriously ill children with providers in remote communities may help to improve skills. Improved provider assessments will help strengthen the health system in Botswana and other resource-limited countries.

### Limitations

The major study design limitation was the lack of a gold standard (such as operational performance or patient outcome) to measure against, which limits our ability to provide evidence linking to other variables. Additionally, the manikin used for simulation is designed for infant cardiopulmonary resuscitation, making it low-fidelity to the scenarios in which it was used, and this may have limited reliability in the assessments of tasks performed in the scenarios. A higher fidelity manikin designed to discriminate performance of tasks for Saving Children’s Lives, such as the manikins used for perinatal resuscitation courses [33–35], may greatly improve assessment reliability for these tasks.

Due to factors extrinsic to the study, we employed a narrow assessment plan of a single case for each participant. As participants did not complete multiple scenarios, we were limited in our ability to examine internal structure. Using only raters familiar with the tool may have restricted the impact of the study, and future studies should demonstrate evidence with instructors unfamiliar with the tool. Finally, we were limited in our analysis due to lower than anticipated enrollment, which may have under-powered our results.

### Future directions

Future studies should be conducted and employ a greater number of participants, additional local raters, and raters who are unfamiliar with the assessment tool. Psychometric data should be used to refine cases and specific items across cases that had less reliability. Development of a low cost, high fidelity manikin may increase the item reliability and assessment of providers. Development of a mobile-based platform may improve the sustainability and scalability of remote simulation skills assessments. Cases could also be conducted on a regular basis to evaluate if practice and repeated exposure improves provider performance in simulated scenarios.

## Conclusion

This study provides preliminary validity evidence of a structured scoring assessment of provider skills in caring for seriously ill children in the clinic environment in LMICs. This study’s strength lies in its ability to provide validity evidence in regards to content, response process, internal structure, and consequences, but is limited in relationships to other variables. Local and international raters evaluate participants similarly for some but not all cases. Remote simulation skills assessment of clinic-based providers in Botswana is reliable and likely feasible, but a low-cost, high-fidelity manikin is needed to improve the reliability of provider assessment. Future studies should include more participants, improved equipment, and utilize additional local raters and raters unfamiliar with the assessment tool.

## Supporting information

S1 Appendix Case 1—1 year old with Acute Respiratory Distress from Lower Respiratory Tract Infection (LRTI). (DOCX)

S2 Appendix Case 2—1 year old with Acute Respiratory Distress from Lower Airway Obstruction (LAO). (DOCX)

S3 Appendix Case 3—1 year old with Shock (hypotensive, hypovolemic) from Severe Dehydration (HSSD). (DOCX)

S4 Appendix Case 4—1 year old with Shock (distributive, sepsis) and Respiratory Distress from Pneumonia (LRTI+DSS). (DOCX)

S5 Appendix SCL Critical Skills Checklist. (DOCX)

S6 Appendix Assessment tools. (DOCX)

S7 Appendix Medication master list. (DOCX)

S8 Appendix Standardized video angles. (DOCX)

S9 Appendix Standard scenario script. (DOCX)

S1 Dataset (XLSX)
