# Supplementary material for: Feasibility and preliminary validity evidence for remote video-based assessment of clinicians in a global health setting
Source: PLoS One. 2019 Aug 2;14(8):e0220565. doi: 10.1371/journal.pone.0220565 (PMC6677291; doi:10.1371/journal.pone.0220565)
Supplement: S1 Appendix — (DOCX) [file pone.0220565.s001.docx]

## Appendix S1: Case 1 – 1 year old with Acute Respiratory Distress from Lower Respiratory Tract Infection (LRTI)

*Write clinic-specific* ***Supply list****:* Stethoscope, blood pressure cuff, pulse oximeter, thermometer, oxygen supplies, IV supplies, syringes

*Complete Informed Consent form, assign study ID number, fill out Participant Information form*

*Read Standard Scenario Script*

*Start video recording, show supply list in front of camera*

*Read*: The Patient is a 1 year old named Ntebo whose mother brought her to you as she has been having difficulty breathing. The child is previously healthy, is HIV(-), and weighs 10 kg.

The triage nurse tells you the initial assessment of the child is eyes open, difficulty breathing, and lips are bluish. [*START 5 MINUTE TIMER*]

Initial State: Temp 38.5, HR 150, RR 50, BP 90/50 pulse Ox: 85%

**Tasks: Assessment (verbalizes patient with lung tissue disease/pneumonia), Applies monitors, Oxygen Therapy, Vascular Access (IV), Medication (Antibiotics), Reassessment, Arrange transport**

**Transport arrives, Hypoxia Resolved, no change in respiratory distress:**

Temp 38, HR 150, RR 50, BP 95/50 pulse Ox: 95%

*Critical to Move on:*

**Oxygen Therapy**

*If IV access attempted*, Instructor responds “IV is successfully placed”

*If oral medications attempted*, Instructor responds “the patient coughs and gets agitated, does not take oral medications”

*When the alarm sounds after 5 minutes,* ***ask***: "Is there anything else you would like to do?"
