# Supplementary material for: Feasibility and preliminary validity evidence for remote video-based assessment of clinicians in a global health setting
Source: PLoS One. 2019 Aug 2;14(8):e0220565. doi: 10.1371/journal.pone.0220565 (PMC6677291; doi:10.1371/journal.pone.0220565)
Supplement: S2 Appendix — (DOCX) [file pone.0220565.s002.docx]

## Appendix S2: Case 2 – 1 year old with Acute Respiratory Distress from Lower Airway Obstruction (LAO)

*Write clinic-specific* ***Supply list****:* Stethoscope, blood pressure cuff, pulse oximeter, thermometer, oxygen supplies, IV supplies, syringes, nebulizer

*Complete Informed Consent form, assign study ID number, fill out Participant Information form*

*Read Standard Scenario Script*

*Start video recording, show supply list in front of camera*

*Read*: The Patient is a 1 year old named Segolame whose mother brought him to you as he has been having difficulty breathing. The child is previously healthy, is HIV(-), and weighs 10 kg. The triage nurse tells you the initial assessment of the child is awake, difficulty breathing, and lips do not appear bluish [*START 5 MINUTE TIMER*]

Initial State: Temp 37, HR 150, RR 50, BP 90/50 pulse Ox: 92%

**Tasks: Assessment (verbalizes patient with lower airway obstruction), applies monitors, Nebulizer therapy, Oxygen therapy, Vascular Access (IV), Medication (steroids), Reassessment, Arrange transport**

**Transport arrives, Resp Distress improved:**

Temp 38, HR 140, RR 30, BP 95/50 pulse Ox: 98%

*Critical to Move on:*

**Albuterol Nebulizer**

*If IV access attempted*, Instructor responds “IV is successfully placed”

*If oral medications attempted*, Instructor responds “the patient coughs and gets agitated, does not take oral medications”

*When the alarm sounds after 5 minutes,* ***ask***: "Is there anything else you would like to do?"
