# Supplementary material for: Feasibility and preliminary validity evidence for remote video-based assessment of clinicians in a global health setting
Source: PLoS One. 2019 Aug 2;14(8):e0220565. doi: 10.1371/journal.pone.0220565 (PMC6677291; doi:10.1371/journal.pone.0220565)
Supplement: S3 Appendix — (DOCX) [file pone.0220565.s003.docx]

## Appendix S3: Case 3 – 1 year old with Shock (hypotensive/hypovolemic) from Severe Dehydration (HSSD)

*Write clinic-specific* ***Supply list****:* Stethoscope, blood pressure cuff, pulse oximeter, thermometer, oxygen supplies, IV supplies, syringes, IO, fluid bag

*Complete Informed Consent form, assign study ID number, fill out Participant Information form*

*Read Standard Scenario Script*

*Start video recording, show supply list in front of camera*

*Read*: The Patient is a 1 year old named Joyce whose mother brought her to you as she has been having vomiting and diarrhea. The child is previously healthy, is HIV(-), and weighs 10 kg. The triage nurse tells you the initial assessment of the child is eyes closed, breathing without difficulty, and not lips do not appear bluish [*START 5 MINUTE TIMER*]

Initial State: Temp 37.5, HR 190, RR 40, BP 65/35 pulse Ox: 95%

**Tasks: Assessment (verbalizes hypotensive hypovolemic shock/severe dehydration), Applies monitors, Oxygen Therapy, IO placement, fluid bolus, Reassessment, arrange transport**

**Transport arrives, Resolving Shock:**

Temp 37.5, HR 150, RR 45, BP 80/50 pulse Ox: 95%

*Critical to Move on:*

**Places IO**

**Gives correct bolus**

*If IV access attempted*, Instructor responds “IV is NOT successfully placed after 3 attempts”

*If oral medications attempted*, Instructor responds “the patient coughs, does not take oral medications”

*When the alarm sounds after 5 minutes,* ***ask***: "Is there anything else you would like to do?"
