# Supplementary material for: Feasibility and preliminary validity evidence for remote video-based assessment of clinicians in a global health setting
Source: PLoS One. 2019 Aug 2;14(8):e0220565. doi: 10.1371/journal.pone.0220565 (PMC6677291; doi:10.1371/journal.pone.0220565)
Supplement: S4 Appendix — (DOCX) [file pone.0220565.s004.docx]

## Appendix S4: Case 4 – 1 year old with Shock (Distributive, Sepsis) and Respiratory Distress from Pneumonia (LRTI+DSS)

*Write clinic-specific* ***Supply list****:* Stethoscope, blood pressure cuff, pulse oximeter, thermometer, oxygen supplies, IV supplies, syringes, IO, fluid bag

*Complete Informed Consent form, assign study ID number, fill out Participant Information form*

*Read Standard Scenario Script*

*Start video recording, show supply list in front of camera*

*Read*: The Patient is a 1 year old named Ruth whose mother reports she has been having difficulty breathing for 3 days and not eating well for 1 day. The child is previously healthy, is HIV(-), and weighs 10 kg. The triage nurse tells you the initial assessment of the child is eyes closed, difficulty breathing, and lips appear bluish. [*START 5 MINUTE TIMER*]

Initial State: Temp 38.5, HR 190, RR 50, BP 70/40 pulse Ox: 85%

**Tasks: Assessment/monitors (Verbalizes compensated distributive shock and lung tissue disease/LRTI), Oxygen, IO placement, Medications (antibiotics), fluid bolus, Reassessment, arrange transport**

**Transport arrives, Hypoxia Resolved and Resolving Shock:**

Temp 38, HR 150, RR 50, BP 90/50 pulse Ox: 95%

*Critical to Move on:*

**Oxygen administration**

**Places IO**

**Gives Fluid Bolus**

*If IV access attempted*, Instructor responds “IV is NOT successfully placed after 3 attempts”

*If oral medications attempted*, Instructor responds “the patient coughs, does not take oral medications”

*When the alarm sounds after 5 minutes,* ***ask***: "Is there anything else you would like to do?"
