# Supplementary figures and images for: Feasibility and preliminary validity evidence for remote video-based assessment of clinicians in a global health setting
Source: PLoS One. 2019 Aug 2;14(8):e0220565. doi: 10.1371/journal.pone.0220565 (PMC6677291; doi:10.1371/journal.pone.0220565)

Appendix S5: SCL Critical Skills Checklist


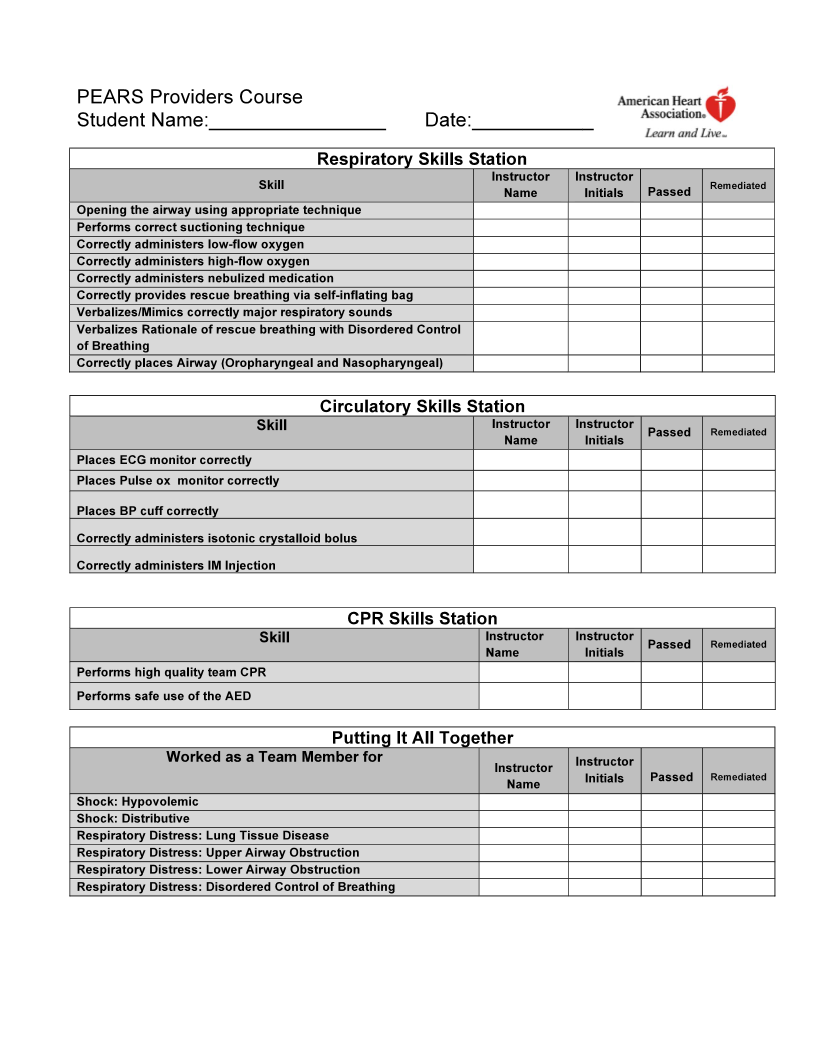

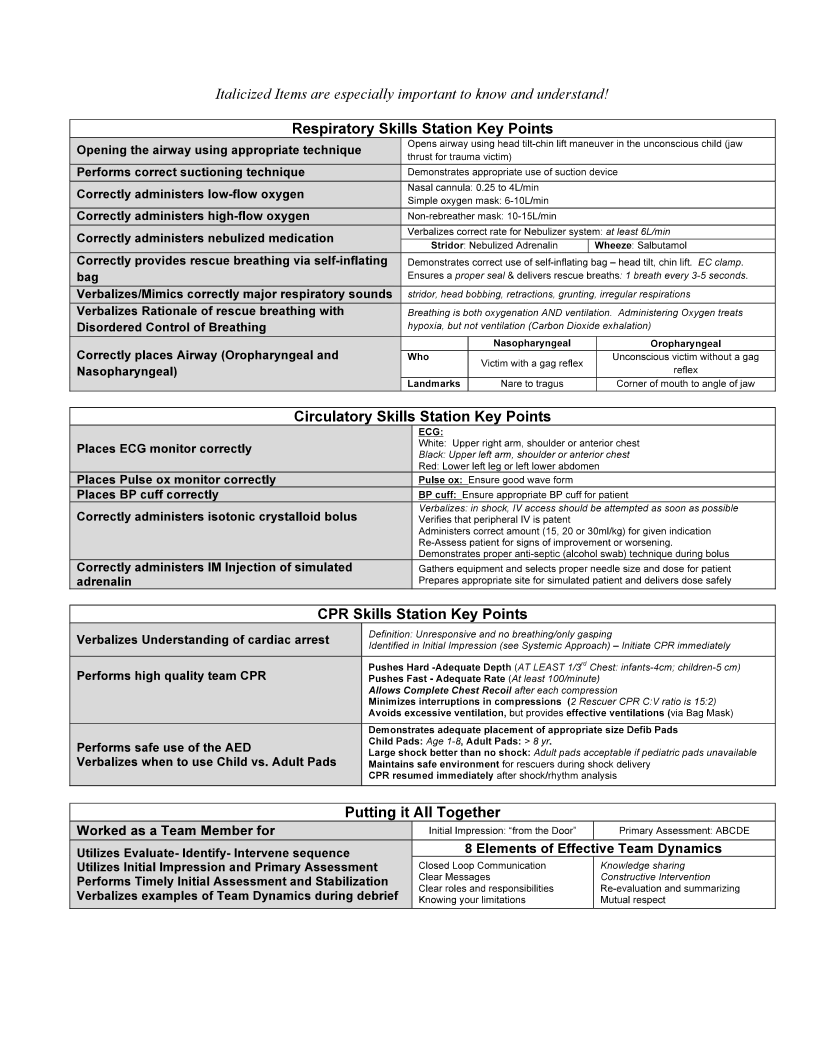

Supplement: S5 Appendix — (DOCX) [file pone.0220565.s005.docx]

## Appendix S6: Assessment Tools


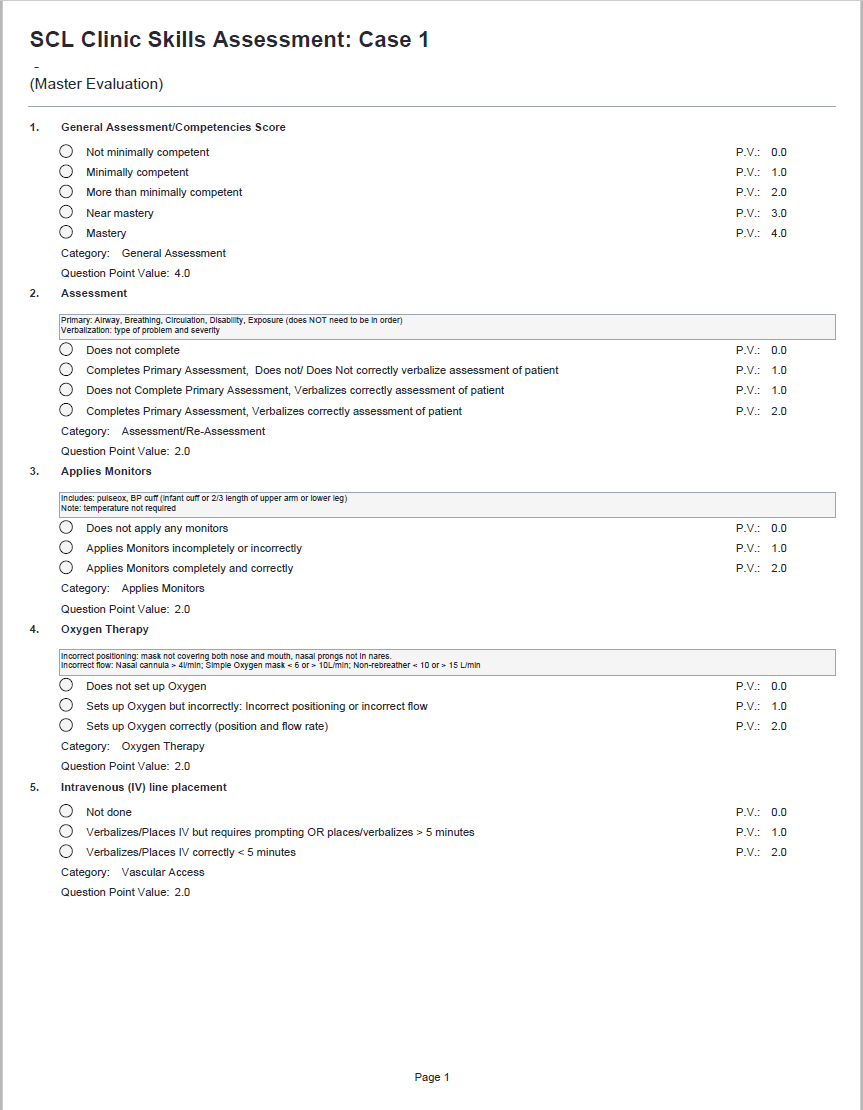

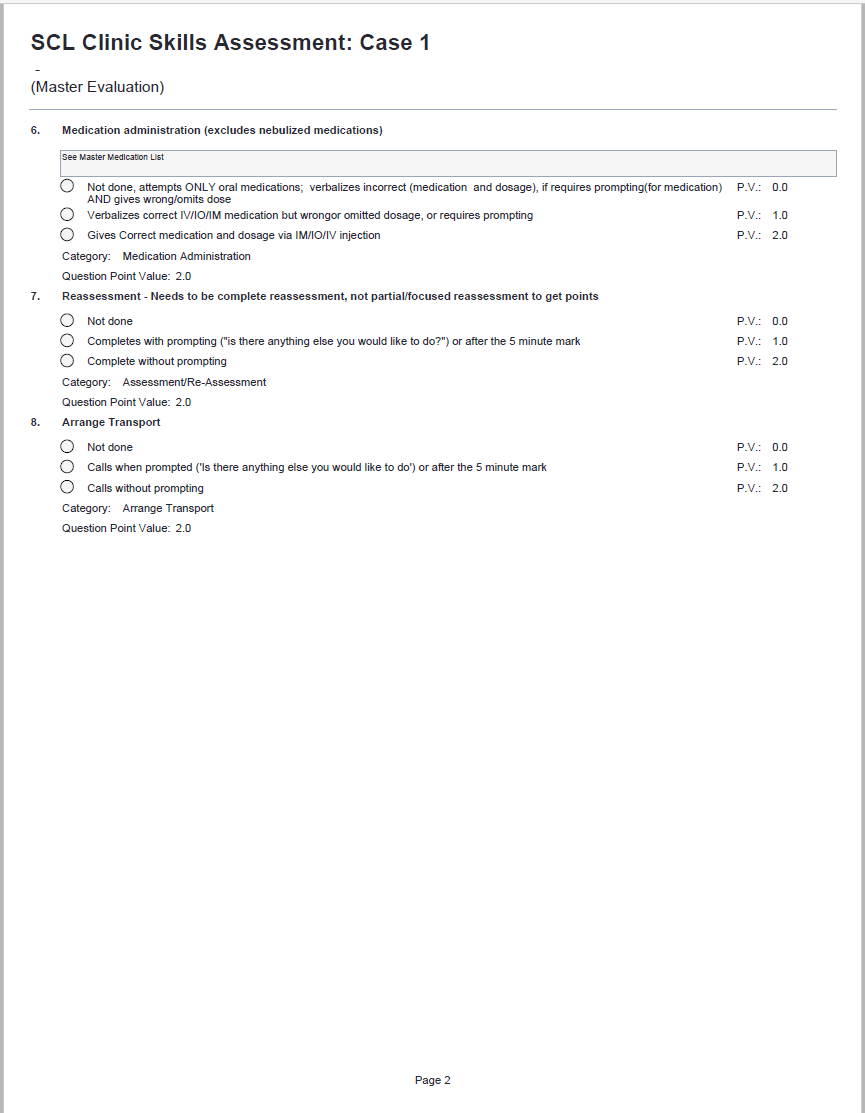


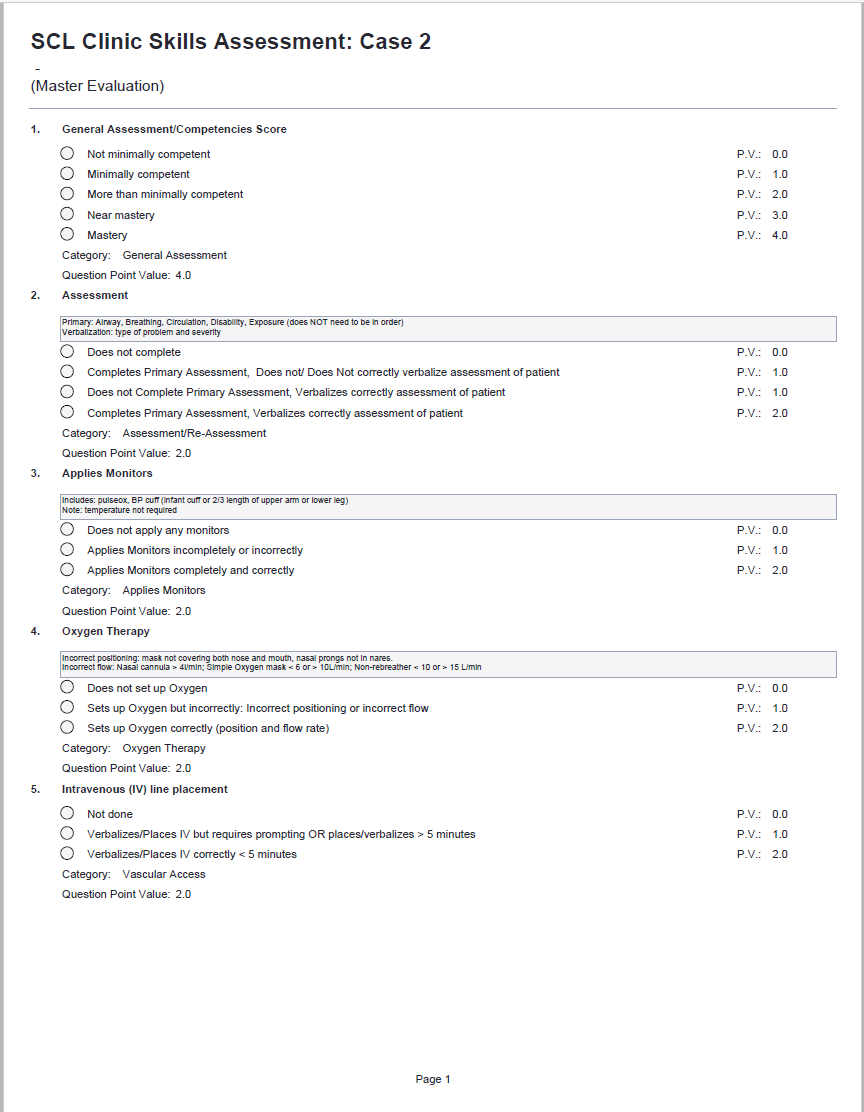

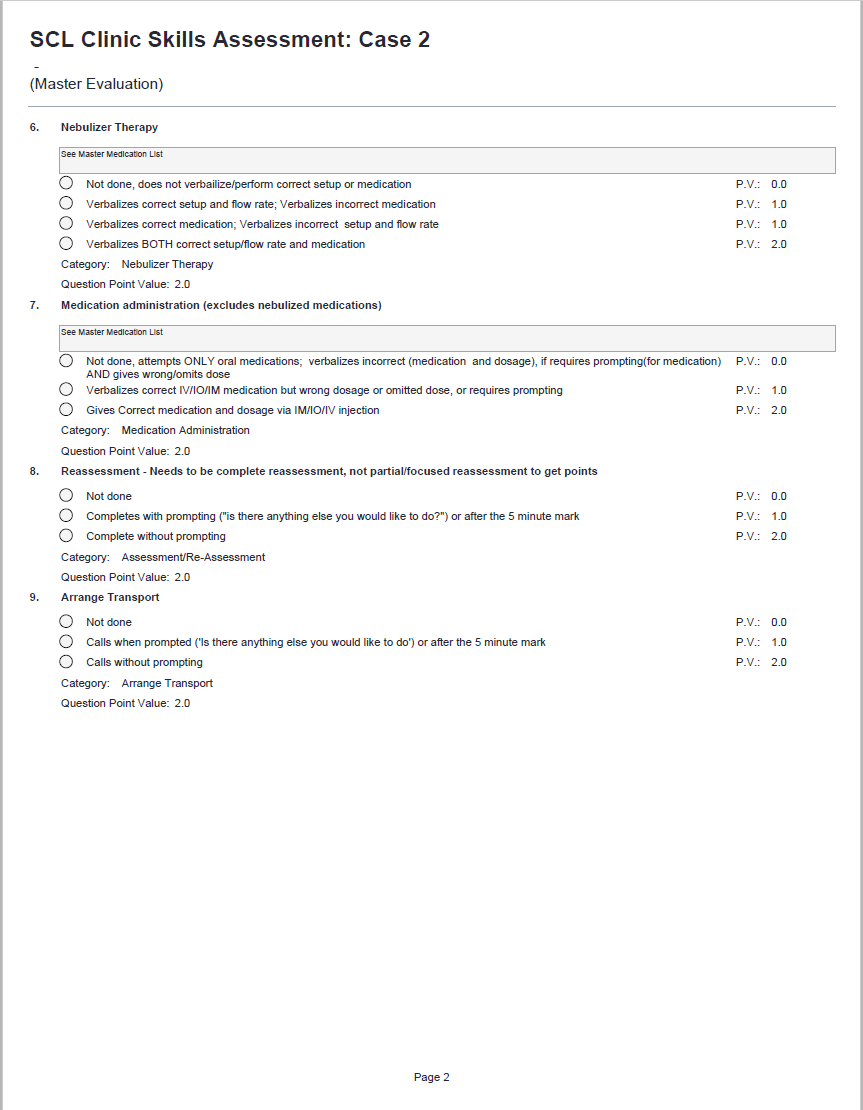


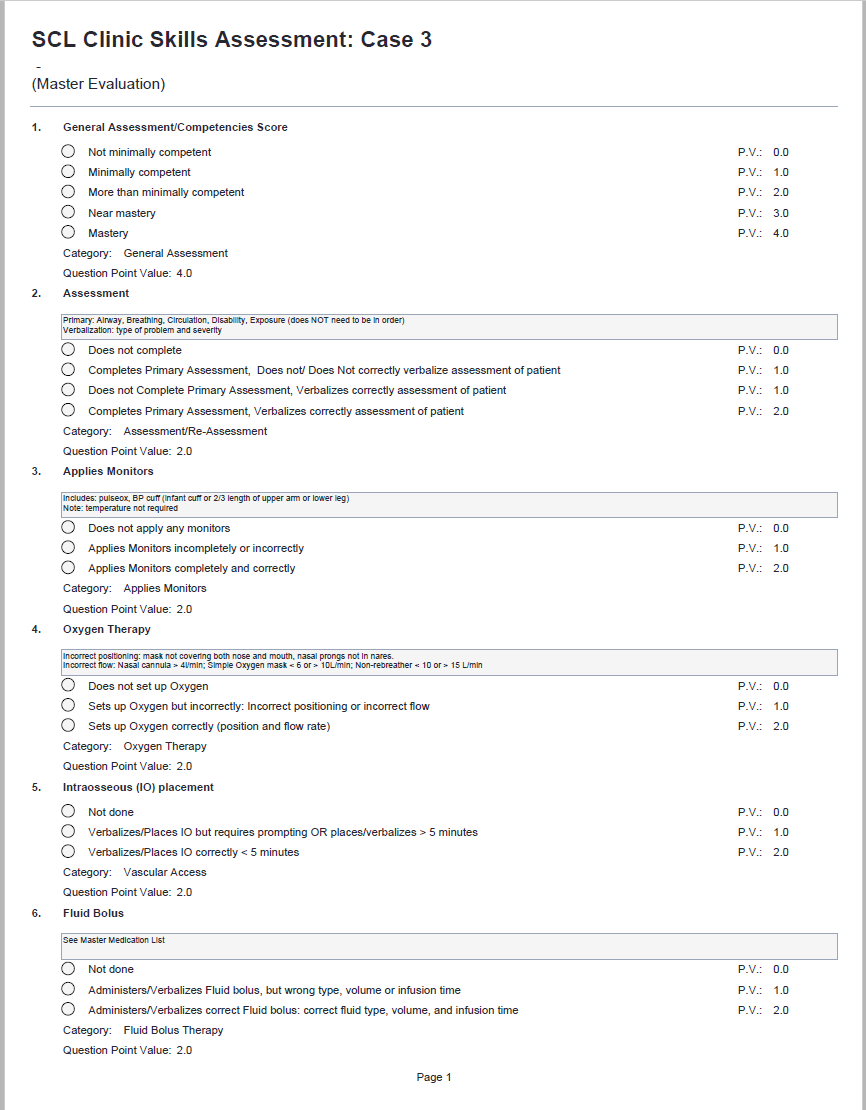

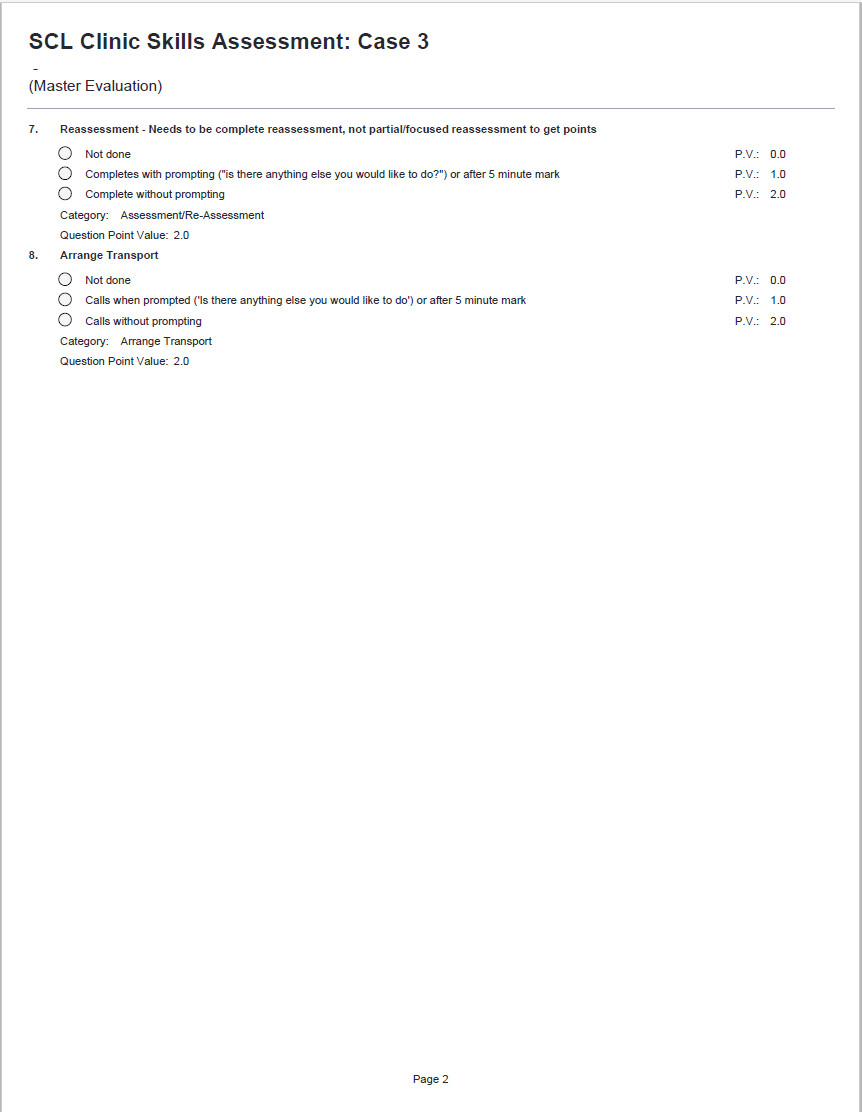


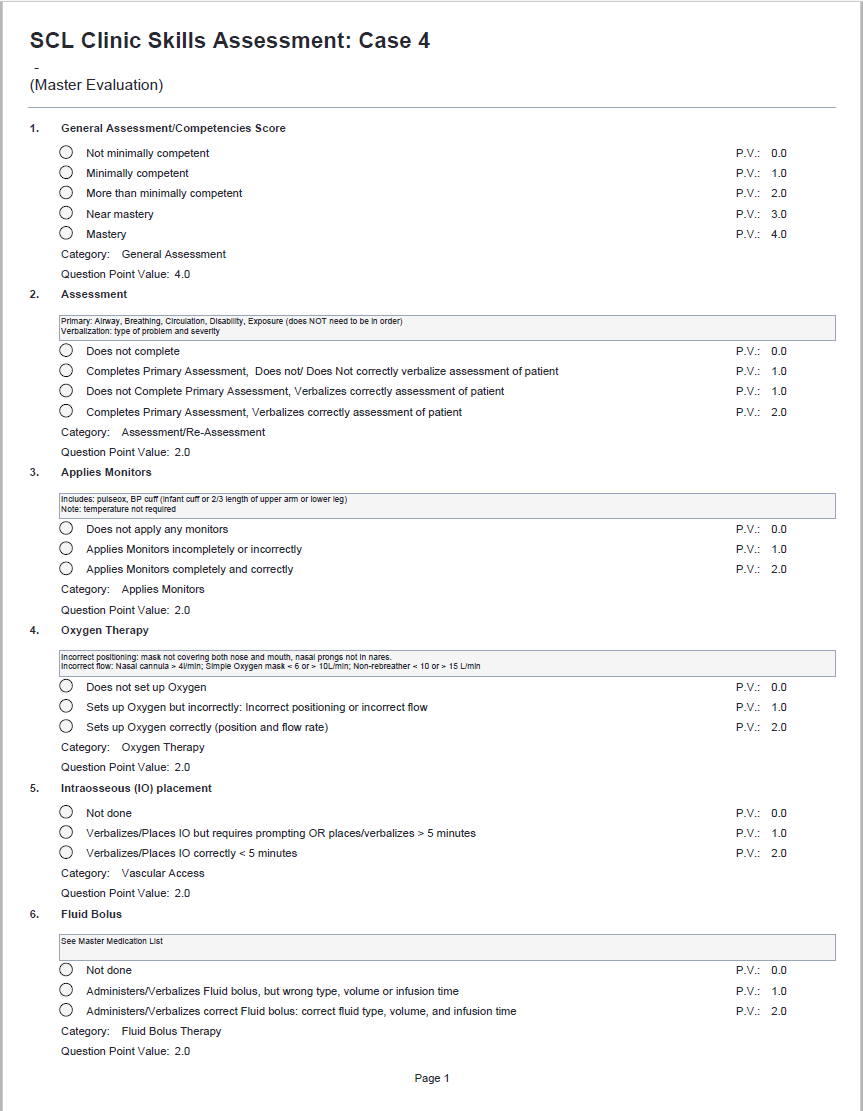

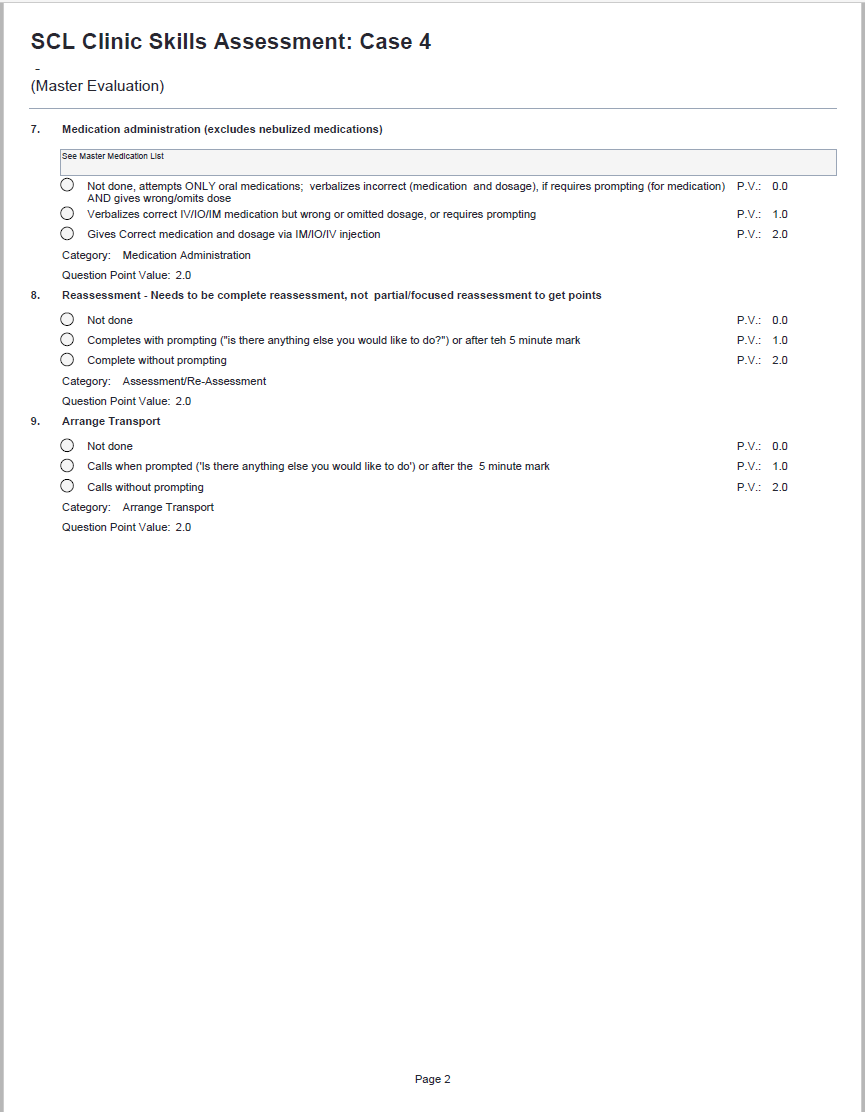

Supplement: S6 Appendix — (DOCX) [file pone.0220565.s006.docx]

## Appendix S7: Medication Master List


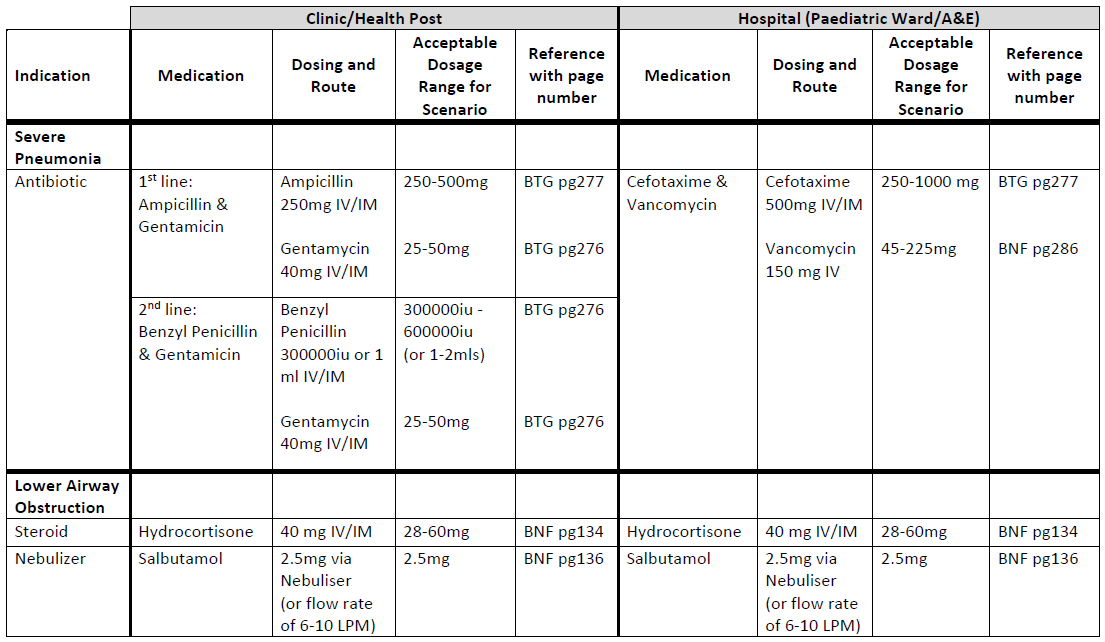


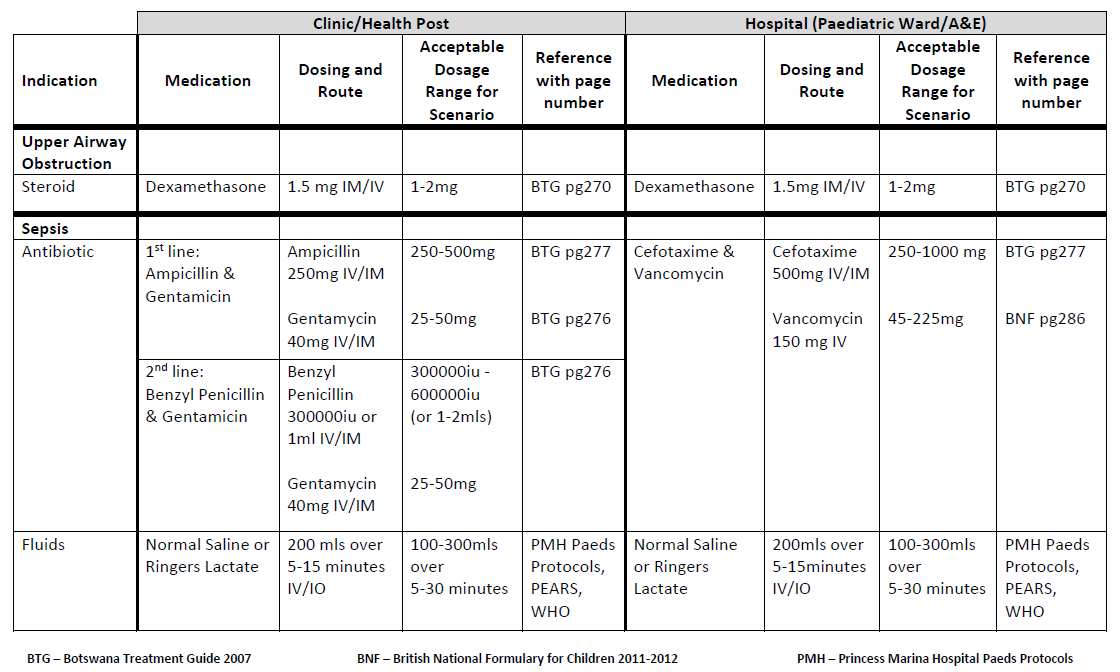

Supplement: S7 Appendix — (DOCX) [file pone.0220565.s007.docx]

## **Appendix S8:** Standardized Video Angles


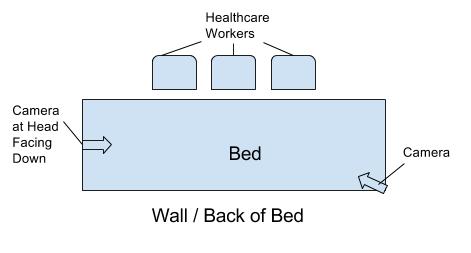


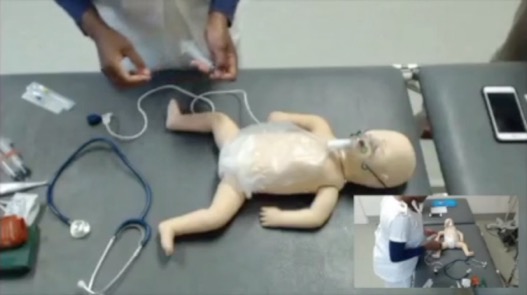

Supplement: S8 Appendix — (DOCX) [file pone.0220565.s008.docx]
