# Supplementary material for: Feasibility and preliminary validity evidence for remote video-based assessment of clinicians in a global health setting
Source: PLoS One. 2019 Aug 2;14(8):e0220565. doi: 10.1371/journal.pone.0220565 (PMC6677291; doi:10.1371/journal.pone.0220565)
Supplement: S9 Appendix — (DOCX) [file pone.0220565.s009.docx]

## Appendix S9: **Standard Scenario Script**

At the Beginning of the scenario: *(Operator sets up the SimCapture)*

Before we begin, we need to complete a consent form that confirms your participation is voluntary.

[*Complete consent form,* ***confirm Study ID is written on consent form and in B line recording notes, and that appropriate scenario is conducted based on Block Randomization Schedule in protocol***]

We also need to gather a little bit of information from you that will remain confidential and separate from this video. [*Complete participant information form, to be entered into excel spreadsheet*]

This assessment is of your skills to assess a seriously ill child. Treat it like a real patient encounter. You should do whatever you think is necessary to evaluate and treat the child. You will have to determine what is needed for the child as well as do it.

- The manikin does not move, respond, breathe, change color, or have a pulse.
- If you choose to do an assessment or a reassessment, please speak clearly about what you are doing and what you are looking for, and I will tell you what you see. For example, if you want to check the breath sounds, put on the stethoscope, place the bell on the part of the chest you want to listen to, and say out loud what you are listening for.
- There is no flow in the oxygen devices or suction catheters. Please verbalize at what flow rate you would like the equipment to be set.
- If you give medications, please state the medication, route, and dose clearly. If you give fluid, place the cannula into the water bottle container and give the amount of fluid.
- I may ask you a question or I may ask you to verbalize your assessment of patient during the scenario, please answer as best you can, but continue the scenario.
- If you make a mistake or forget to do something important, you should not stop. Just do your best to correct the error. Please continue doing what you would do in an actual emergency until I tell you to stop.
- The Scenario is 5 minutes long and will time out at the conclusion, when you will hear an audible alarm sound. Please accomplish as many things as you can in that time frame.
- You may use any of the provided supplies to complete the scenario, even if you do not have them available at your facility.

Do you have any questions before we start?

- I am going to tell some information about the patient and then we will start.
- *(Operator starts B-line session, show clinic-specific supply list to camera)*
- *Facilitator/Instructor read the case description*
- Other questions about the child you can ask me after the Assessment starts.
- The Assessment starts now. [*START 5 MINUTE TIMER*]

At the Conclusion of the scenario, when the alarm sounds:

Please stop, the Scenario is over. Is there anything else you would have done or continued if you had more time?

Great Job! Now we are going to debrief about what you did.

*(Proceed with 10 minutes debriefing with the instructor / instructor candidate)*

This concludes the debriefing. Thank you for participating. *(Operator ends B-line recording)*

**Once back at SLH:**

- Scan consent form into Dropbox and file hard copy in locked cabinet
- Enter participant information into password-protected excel spreadsheet
- Lock computer and connect to network to allow video upload to B line overnight
